# *catena*-Poly[[manganese(II)-tris­(μ-bet­aine-κ^2^ *O*:*O*′)] tetra­bromido­manganate(IV)

**DOI:** 10.1107/S1600536808028912

**Published:** 2008-09-17

**Authors:** Maria Kocadag, Michel Fleck, Ladislav Bohatý

**Affiliations:** a Universität Wien – Geozentrum, Institut für Mineralogie und Kristallographie, Althanstrasse 14, A-1090 Wien, Austria; b Universität zu Köln, Institut für Kristallographie, Zülpicher Strasse 49b, D-50674 Köln, Germany

## Abstract

The title compound, [Mn(C_5_H_11_NO_2_)_3_]·MnBr_4_, contains polymeric cationic chains of distorted MnO_6_ octa­hedra and bridging betaine mol­ecules, running parallel to the *a* axis. There are two distinct Mn^2+^ cations in the chain, both with site symmetry 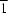. Distorted [MnBr_4_]^2−^ tetra­hedra occupy the spaces between the chains.

## Related literature

For related literature, see: Chen & Mak (1994 ▶); Haussühl (1988 ▶, 1989 ▶); Haussühl & Schreuer (2001 ▶); Haussühl & Wang (1989 ▶); Mak (1990 ▶); Viertorinne *et al.* (1999 ▶); Wang *et al.* (1986 ▶); Wiehl *et al.* (2006*a* ▶,*b* ▶)); Chen & Mak (1991 ▶); Schreuer & Haussühl (1993 ▶).
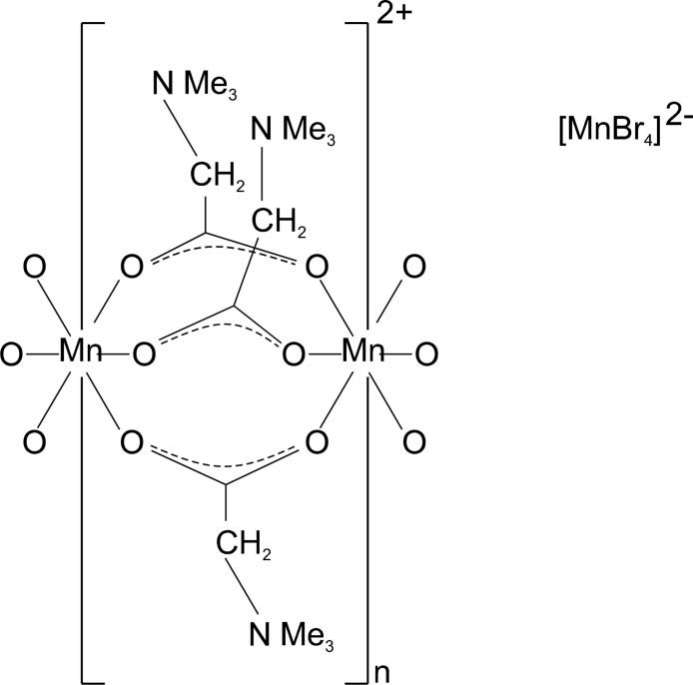


## Experimental

### 

#### Crystal data

[Mn(C_5_H_11_NO_2_)_3_]·MnBr_4_
*M* *_r_* = 780.96
Triclinic, 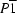

*a* = 9.140 (2) Å
*b* = 12.700 (2) Å
*c* = 12.871 (3) Å
α = 66.557 (6)°
β = 86.063 (7)°
γ = 89.249 (7)°
*V* = 1367.3 (4) Å^3^
*Z* = 2
Mo *K*α radiation
μ = 6.80 mm^−1^
*T* = 293 (2) K
0.50 × 0.30 × 0.30 mm

#### Data collection

Bruker SMART CCD diffractometer
Absorption correction: multi-scan (Otwinowski & Minor, 1997 ▶) *T*_min_ = 0.073, *T*_max_ = 0.130
17423 measured reflections
7836 independent reflections
5085 reflections with *I* > 2σ(*I*)
*R*_int_ = 0.029

#### Refinement

*R*[*F* ^2^ > 2σ(*F* ^2^)] = 0.053
*wR*(*F* ^2^) = 0.115
*S* = 1.05
7836 reflections
326 parameters
H atoms treated by a mixture of independent and constrained refinement
Δρ_max_ = 1.77 e Å^−3^
Δρ_min_ = −1.88 e Å^−3^

### 

Data collection: *SMART* (Bruker, 2004 ▶); cell refinement: *SMART*; data reduction: *SAINT* (Bruker, 2004 ▶); program(s) used to solve structure: *SHELXS97* (Sheldrick, 2008 ▶); program(s) used to refine structure: *SHELXL97* (Sheldrick, 2008 ▶); molecular graphics: *DIAMOND* (Bergerhoff *et al.*, 1996 ▶); software used to prepare material for publication: *SHELXL97*.

## Supplementary Material

Crystal structure: contains datablocks global, I. DOI: 10.1107/S1600536808028912/hb2779sup1.cif

Structure factors: contains datablocks I. DOI: 10.1107/S1600536808028912/hb2779Isup2.hkl

Additional supplementary materials:  crystallographic information; 3D view; checkCIF report

## Figures and Tables

**Table 1:** Selected bond lengths (Å)

Mn1—O2*C*	2.173 (3)
Mn1—O1*B*	2.176 (3)
Mn1—O1*A*^i^	2.219 (3)
Mn2—O1*C*	2.131 (3)
Mn2—O2*B*	2.163 (3)
Mn2—O2*A*	2.192 (3)
Mn3—Br2	2.4724 (11)
Mn3—Br4	2.4932 (11)
Mn3—Br1	2.5019 (12)
Mn3—Br3	2.5179 (11)

Symmetry code: (i) 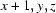.

**Fig. 1.**
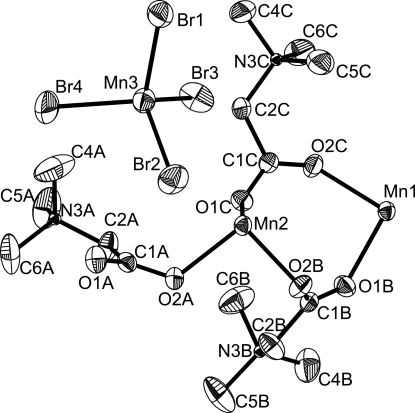
The asymmetric unit of (I), shown with displacement ellipsoids at the 50% probability level. Hydrogen atoms are omitted for clarity.

**Fig. 2.**
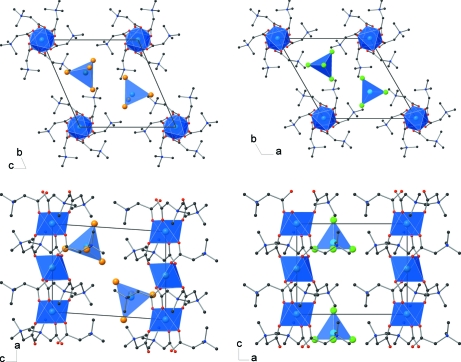
Packing diagram for (I) (left) in comparison with the structure of ([(betaine)3Mn](MCl4)] (Wiehl et al., 2006a, right), viewed along and perpendicular to the chains (top and bottom). Hydrogen atoms are omitted for clarity.

## supplementary crystallographic information

### Comment

The physical properties of compounds of metal salts with the zwitterionic ligand betaine have been investigated intensively in the past (Haussühl & Schreuer, 2001; Haussühl & Wang, 1989; Haussühl, 1989; Haussühl, 1988; Wang *et al.*, 1986; Chen & Mak, 1994, and references therein).

In particular, the low-dimensional magnetic properties of the isomorphic trigonal group of betaine manganese metal chlorides [(C_5_H_11_NO_2_)_3_Mn].*M*Cl_4_ (*M* = Mn, Co, Zn, space group *P*3) has been analysed in detail (Wiehl *et al.*, 2006*b*). In these crystals, the betaine ligands operate as µ-(*O*,*O*') bridges between Mn^2+^ cations thus forming chains of the octahedrally coordinated mangnetic cations (*S* = 5/2). A model of an antiferromagnetic Heisenberg spin fits well the magnetic proerties of these crystals (Wiehl *et al.*, 2006*b*).

Here, we present the crystal structure of the title compound, (I) (Fig. 1). The crystal structure of (I) contains three crystallographically non-equivalent mangenese atoms. Two of them, Mn1 and Mn2, located on centres of inversion, are sixfold coordinated by oxygen atoms of the carboxylate groups of betaine molecules, which act as bridging ligands to form an one-dimensional tris(carboxylato-*O*,*O*')-bridged Mn^2+^ complex (Table 1). The cationic chains are oriented along the *a* axis and possess approximately the rod symmetry *P*3 (Fig. 2). In the interstices between these chains, anionic distorted tetrahedral groups [Mn(3)Br_4_]^2-^ are located.

Apart from the triclinic symmetry, the structural features of (I) are analogous to those of the trigonal chlorides [(C_5_H_11_NO_2_)_3_Mn].*M*Cl_4_ [*M* = Mn (Chen & Mak, 1991; Schreuer & Haussühl, 1993), Co (Wiehl *et al.*, 2006a) and Zn (Wiehl *et al.*, 2006b)]. The type of structural units [(C_5_H_11_NO_2_)_3_Mn]_∞_ and [MnBr_4_], and the structural packing of these chains and tetrahedra are analogous to the atomic arrangement in the structure of the chloride compounds. Both, the translation period in the direction of the chain axis [*a* in (I), *c*≈ 9.08 Å in the chloride compounds] as well as the interchain distances [*b* and *c* in (I), *a* = *b*≈ 12.8 Å in the chloride compounds] correspond well. The interatomic distances and angles within the betaine molecules agree well with the values given in the literature (Viertorinne *et al.*, 1999; Mak, 1990), the carboxylate C—O distances indicate the delocalization of the electrons to a mesomeric state [C—O-distances range from 1.245 (5) to 1.255 (5) Å].

### Experimental

The title compound crystallizes from 3:2 stoichiometry aqueous solutions of betaine and MnBr_2_ in the temperature range 290 to 300 K in thick tabular prismatic crystals of sulfur-yellow colour. Crystals of optical quality with dimensions up to 25 × 7 × 3 mm were grown from a solution of 167 g betaine and 272 g MnBr_2_.4H_2_O by very slow evaporation at 295 K during a period of 11 months.

### Crystal data

**Table:**

[Mn(C_5_H_11_NO_2_)_3_]·MnBr_4_	*Z* = 2
*M**_r_* = 780.96	*F*(000) = 764
Triclinic, *P*1	*D*_x_ = 1.897 Mg m^−^^3^
Hall symbol: -P 1	Melting point: not determined K
*a* = 9.140 (2) Å	Mo *K*α radiation, λ = 0.71073 Å
*b* = 12.700 (2) Å	Cell parameters from 1294 reflections
*c* = 12.871 (3) Å	θ = 2.8–26.4°
α = 66.557 (6)°	µ = 6.80 mm^−^^1^
β = 86.063 (7)°	*T* = 293 K
γ = 89.249 (7)°	Prism, yellow
*V* = 1367.3 (4) Å^3^	0.50 × 0.30 × 0.30 mm

### Data collection

**Table:**

Bruker SMART CCD diffractometer	7836 independent reflections
Radiation source: fine-focus sealed tube	5085 reflections with *I* > 2σ(*I*)
graphite	*R*_int_ = 0.030
Detector resolution: 0.1 pixels mm^-1^	θ_max_ = 30°, θ_min_ = 1.8°
φ–scan and ω–scans	*h* = −13→13
Absorption correction: multi-scan (Otwinowski & Minor, 1997)	*k* = −18→18
*T*_min_ = 0.073, *T*_max_ = 0.130	*l* = −15→18
17423 measured reflections	

### Refinement

**Table:**

Refinement on *F*^2^	Secondary atom site location: difference Fourier map
Least-squares matrix: full	Hydrogen site location: inferred from neighbouring sites
*R*[*F*^2^ > 2σ(*F*^2^)] = 0.054	H atoms treated by a mixture of independent and constrained refinement
*wR*(*F*^2^) = 0.115	*w* = 1/[σ^2^(*F*_o_^2^) + (0.0151*P*)^2^ + 6.4946*P*] where *P* = (*F*_o_^2^ + 2*F*_c_^2^)/3
*S* = 1.05	(Δ/σ)_max_ < 0.001
7836 reflections	Δρ_max_ = 1.77 e Å^−^^3^
326 parameters	Δρ_min_ = −1.88 e Å^−^^3^
0 restraints	Extinction correction: SHELXL97 (Sheldrick, 2008), Fc^*^=kFc[1+0.001xFc^2^λ^3^/sin(2θ)]^-1/4^
Primary atom site location: structure-invariant direct methods	Extinction coefficient: 0.0015 (3)

### Special details

**Table:**

Experimental. Single-crystal X-ray intensity data were collected at 293 K on a Nonius APEXII diffractometer with CCD-area detector, using 673 frames with phi- and omega-increments of 1 degree and a counting time of 60 s per frame. The crystal-to-detector-distance was 30 mm. The whole ewald sphere was measured. The reflection data were processed with the Nonius program suite *DENZO-SMN* and corrected for Lorentz, polarization, background and absorption effects (Otwinowski and Minor, 1997). The crystal structure was determined by Direct methods (*SHELXS97*, Sheldrick, 2008) and subsequent Fourier and difference Fourier syntheses, followed by full-matrix least-squares refinements on F^2^ (*SHELXL97*, Sheldrick, 2008). All hydrogen atoms were treated as riding. Using anisotropic treatment of the non-H atoms and unrestrained isotropic treatment of the H atoms, the refinement converged at an *R*-value of 0.053.
Geometry. All e.s.d.'s (except the e.s.d. in the dihedral angle between two l.s. planes) are estimated using the full covariance matrix. The cell e.s.d.'s are taken into account individually in the estimation of e.s.d.'s in distances, angles and torsion angles; correlations between e.s.d.'s in cell parameters are only used when they are defined by crystal symmetry. An approximate (isotropic) treatment of cell e.s.d.'s is used for estimating e.s.d.'s involving l.s. planes.
Refinement. Refinement of *F*^2^ against ALL reflections. The weighted *R*-factor *wR* and goodness of fit *S* are based on *F*^2^, conventional *R*-factors *R* are based on *F*, with *F* set to zero for negative *F*^2^. The threshold expression of *F*^2^ > σ(*F*^2^) is used only for calculating *R*-factors(gt) *etc*. and is not relevant to the choice of reflections for refinement. *R*-factors based on *F*^2^ are statistically about twice as large as those based on *F*, and *R*- factors based on ALL data will be even larger.

### Fractional atomic coordinates and isotropic or equivalent isotropic displacement parameters (Å^2^)

**Table:**

	*x*	*y*	*z*	*U*_iso_*/*U*_eq_	
Mn1	1.0000	0.0000	1.0000	0.0226 (2)	
Mn2	0.5000	0.0000	1.0000	0.0234 (2)	
Mn3	0.77120 (8)	0.62332 (7)	0.69412 (7)	0.0388 (2)	
Br1	0.72670 (6)	0.62971 (5)	0.88548 (5)	0.04926 (17)	
Br2	0.73289 (7)	0.43013 (5)	0.69615 (7)	0.05729 (19)	
Br3	1.03439 (7)	0.68376 (6)	0.62887 (6)	0.05688 (19)	
Br4	0.60829 (11)	0.76456 (8)	0.56062 (8)	0.0922 (3)	
O1A	0.1462 (3)	0.1052 (3)	0.8505 (3)	0.0338 (8)	
O2A	0.3773 (3)	0.0449 (3)	0.8480 (3)	0.0301 (7)	
C1A	0.2801 (4)	0.1200 (4)	0.8221 (4)	0.0256 (9)	
C2A	0.3415 (5)	0.2387 (4)	0.7460 (6)	0.0372 (12)	
H2A1	0.426 (6)	0.258 (5)	0.781 (5)	0.046 (16)*	
H2A2	0.375 (7)	0.234 (5)	0.682 (6)	0.06 (2)*	
N3A	0.2438 (4)	0.3404 (3)	0.7206 (4)	0.0366 (10)	
C4A	0.1914 (11)	0.3524 (6)	0.8271 (7)	0.082 (3)	
H4A1	0.1203	0.4122	0.8106	0.10 (3)*	
H4A2	0.2728	0.3715	0.8600	0.13 (4)*	
H4A3	0.1472	0.2813	0.8797	0.11 (3)*	
C5A	0.3320 (7)	0.4447 (5)	0.6441 (8)	0.068 (2)	
H5A1	0.3296	0.4528	0.5668	0.17 (5)*	
H5A2	0.4316	0.4364	0.6650	0.11 (3)*	
H5A3	0.2912	0.5116	0.6515	0.09 (3)*	
C6A	0.1167 (7)	0.3324 (6)	0.6576 (7)	0.067 (2)	
H6A1	0.0802	0.4078	0.6166	0.08 (2)*	
H6A2	0.0406	0.2857	0.7104	0.10 (3)*	
H6A3	0.1474	0.2984	0.6053	0.14 (4)*	
O1B	0.8835 (3)	−0.0491 (3)	0.8839 (3)	0.0326 (7)	
O2B	0.6524 (3)	−0.1059 (3)	0.9500 (3)	0.0333 (7)	
C1B	0.7526 (4)	−0.0671 (3)	0.8728 (4)	0.0265 (9)	
C2B	0.7027 (6)	−0.0439 (5)	0.7562 (5)	0.0423 (13)	
H2B1	0.718 (5)	−0.112 (5)	0.743 (5)	0.036 (14)*	
H2B2	0.601 (8)	−0.024 (6)	0.750 (7)	0.08 (2)*	
N3B	0.7830 (4)	0.0477 (3)	0.6558 (4)	0.0329 (9)	
C4B	0.9394 (6)	0.0214 (7)	0.6382 (6)	0.0612 (19)	
H4B1	0.9462	−0.0541	0.6382	0.11 (3)*	
H4B2	0.9934	0.0246	0.6983	0.10 (3)*	
H4B3	0.9798	0.0767	0.5667	0.08 (2)*	
C5B	0.7081 (8)	0.0607 (7)	0.5518 (6)	0.069 (2)	
H5B1	0.7560	0.1207	0.4868	0.07 (2)*	
H5B2	0.6073	0.0799	0.5602	0.10 (3)*	
H5B3	0.7131	−0.0101	0.5415	0.12 (4)*	
C6B	0.7741 (9)	0.1589 (5)	0.6699 (6)	0.0625 (19)	
H6B1	0.8302	0.2167	0.6083	0.07 (2)*	
H6B2	0.8128	0.1496	0.7405	0.08 (2)*	
H6B3	0.6736	0.1817	0.6701	0.14 (4)*	
O1C	0.6456 (3)	0.1430 (3)	0.9163 (3)	0.0290 (7)	
O2C	0.8693 (3)	0.1519 (3)	0.9718 (3)	0.0323 (7)	
C1C	0.7365 (4)	0.1769 (3)	0.9644 (4)	0.0248 (9)	
C2C	0.6737 (5)	0.2597 (4)	1.0147 (5)	0.0322 (11)	
H2C1	0.668 (6)	0.336 (5)	0.954 (5)	0.041 (15)*	
H2C2	0.579 (5)	0.235 (4)	1.044 (4)	0.030 (13)*	
N3C	0.7558 (4)	0.2747 (3)	1.1054 (4)	0.0313 (9)	
C4C	0.6680 (6)	0.3497 (5)	1.1501 (6)	0.0476 (14)	
H4C1	0.5749	0.3134	1.1829	0.06 (2)*	
H4C2	0.6528	0.4223	1.0891	0.06 (2)*	
H4C3	0.7200	0.3615	1.2070	0.061 (19)*	
C5C	0.7746 (7)	0.1616 (5)	1.2014 (5)	0.0526 (15)	
H5C1	0.8323	0.1127	1.1745	0.062 (19)*	
H5C2	0.6802	0.1265	1.2316	0.08 (2)*	
H5C3	0.8236	0.1731	1.2598	0.07 (2)*	
C6C	0.9027 (6)	0.3312 (5)	1.0602 (6)	0.0526 (16)	
H6C1	0.9568	0.3300	1.1219	0.09 (3)*	
H6C2	0.8897	0.4093	1.0086	0.08 (2)*	
H6C3	0.9557	0.2907	1.0209	0.063 (19)*	

### Atomic displacement parameters (Å^2^)

**Table:**

	*U*^11^	*U*^22^	*U*^33^	*U*^12^	*U*^13^	*U*^23^
Mn1	0.0179 (4)	0.0249 (4)	0.0249 (5)	0.0018 (3)	−0.0021 (3)	−0.0098 (4)
Mn2	0.0181 (4)	0.0239 (4)	0.0285 (6)	0.0012 (3)	−0.0028 (3)	−0.0106 (4)
Mn3	0.0387 (4)	0.0352 (4)	0.0424 (5)	0.0046 (3)	−0.0074 (3)	−0.0146 (4)
Br1	0.0429 (3)	0.0614 (4)	0.0466 (4)	0.0068 (2)	−0.0049 (2)	−0.0247 (3)
Br2	0.0566 (4)	0.0403 (3)	0.0771 (5)	−0.0048 (2)	−0.0039 (3)	−0.0255 (3)
Br3	0.0526 (3)	0.0688 (4)	0.0461 (4)	−0.0236 (3)	0.0062 (3)	−0.0204 (3)
Br4	0.1235 (7)	0.0924 (6)	0.0730 (6)	0.0703 (5)	−0.0529 (5)	−0.0408 (5)
O1A	0.0239 (15)	0.0365 (17)	0.033 (2)	−0.0015 (12)	0.0014 (13)	−0.0057 (15)
O2A	0.0284 (15)	0.0291 (15)	0.032 (2)	0.0063 (12)	−0.0075 (13)	−0.0110 (14)
C1A	0.0254 (19)	0.030 (2)	0.021 (2)	0.0010 (16)	−0.0029 (16)	−0.0093 (18)
C2A	0.024 (2)	0.031 (2)	0.049 (4)	0.0024 (17)	−0.001 (2)	−0.007 (2)
N3A	0.032 (2)	0.0296 (19)	0.043 (3)	0.0016 (15)	−0.0035 (18)	−0.0094 (19)
C4A	0.142 (8)	0.047 (4)	0.059 (5)	0.013 (4)	0.014 (5)	−0.027 (4)
C5A	0.052 (4)	0.031 (3)	0.093 (7)	−0.007 (2)	0.001 (3)	0.005 (3)
C6A	0.043 (3)	0.052 (4)	0.087 (6)	0.010 (3)	−0.031 (3)	−0.004 (4)
O1B	0.0256 (15)	0.0440 (18)	0.033 (2)	0.0013 (13)	−0.0051 (13)	−0.0200 (16)
O2B	0.0291 (16)	0.0305 (16)	0.042 (2)	0.0028 (12)	0.0042 (14)	−0.0171 (15)
C1B	0.025 (2)	0.0241 (19)	0.033 (3)	0.0046 (15)	−0.0050 (17)	−0.0139 (19)
C2B	0.040 (3)	0.046 (3)	0.036 (3)	−0.013 (2)	−0.009 (2)	−0.010 (2)
N3B	0.033 (2)	0.036 (2)	0.029 (2)	0.0030 (16)	−0.0049 (16)	−0.0125 (18)
C4B	0.049 (3)	0.089 (5)	0.037 (4)	0.026 (3)	0.005 (3)	−0.018 (4)
C5B	0.074 (5)	0.077 (5)	0.037 (4)	−0.018 (4)	−0.024 (3)	−0.001 (3)
C6B	0.095 (5)	0.038 (3)	0.050 (5)	0.003 (3)	0.016 (4)	−0.016 (3)
O1C	0.0289 (15)	0.0280 (15)	0.029 (2)	−0.0044 (12)	−0.0020 (13)	−0.0105 (13)
O2C	0.0261 (15)	0.0288 (15)	0.045 (2)	0.0060 (12)	−0.0052 (14)	−0.0168 (15)
C1C	0.0258 (19)	0.0199 (18)	0.026 (3)	−0.0009 (14)	−0.0003 (16)	−0.0070 (17)
C2C	0.021 (2)	0.039 (3)	0.042 (3)	0.0059 (18)	−0.0085 (19)	−0.022 (2)
N3C	0.0264 (18)	0.0337 (19)	0.039 (3)	0.0031 (15)	−0.0048 (16)	−0.0196 (18)
C4C	0.042 (3)	0.059 (4)	0.058 (4)	0.011 (3)	−0.005 (3)	−0.040 (3)
C5C	0.073 (4)	0.044 (3)	0.044 (4)	0.008 (3)	−0.017 (3)	−0.019 (3)
C6C	0.031 (3)	0.060 (4)	0.082 (5)	−0.011 (2)	0.003 (3)	−0.044 (4)

### Geometric parameters (Å, °)

**Table:**

Mn1—O2C^i^	2.173 (3)	O2B—C1B	1.252 (5)
Mn1—O2C	2.173 (3)	C1B—C2B	1.512 (7)
Mn1—O1B	2.176 (3)	C2B—N3B	1.503 (7)
Mn1—O1B^i^	2.176 (3)	C2B—H2B1	0.95 (5)
Mn1—O1A^ii^	2.219 (3)	C2B—H2B2	0.96 (7)
Mn1—O1A^iii^	2.219 (3)	N3B—C4B	1.487 (6)
Mn2—O1C^ii^	2.131 (3)	N3B—C6B	1.493 (7)
Mn2—O1C	2.131 (3)	N3B—C5B	1.495 (7)
Mn2—O2B	2.163 (3)	C4B—H4B1	0.9600
Mn2—O2B^ii^	2.163 (3)	C4B—H4B2	0.9600
Mn2—O2A^ii^	2.192 (3)	C4B—H4B3	0.9600
Mn2—O2A	2.192 (3)	C5B—H5B1	0.9600
Mn3—Br2	2.4724 (11)	C5B—H5B2	0.9600
Mn3—Br4	2.4932 (11)	C5B—H5B3	0.9600
Mn3—Br1	2.5019 (12)	C6B—H6B1	0.9600
Mn3—Br3	2.5179 (11)	C6B—H6B2	0.9600
O1A—C1A	1.247 (5)	C6B—H6B3	0.9600
O2A—C1A	1.255 (5)	O1C—C1C	1.245 (5)
C1A—C2A	1.522 (6)	O2C—C1C	1.251 (5)
C2A—N3A	1.499 (6)	C1C—C2C	1.527 (6)
C2A—H2A1	1.00 (6)	C2C—N3C	1.505 (6)
C2A—H2A2	0.89 (7)	C2C—H2C1	0.97 (6)
N3A—C4A	1.485 (8)	C2C—H2C2	0.92 (5)
N3A—C6A	1.488 (7)	N3C—C5C	1.493 (7)
N3A—C5A	1.502 (7)	N3C—C4C	1.495 (6)
C4A—H4A1	0.9600	N3C—C6C	1.497 (6)
C4A—H4A2	0.9600	C4C—H4C1	0.9600
C4A—H4A3	0.9600	C4C—H4C2	0.9600
C5A—H5A1	0.9600	C4C—H4C3	0.9600
C5A—H5A2	0.9600	C5C—H5C1	0.9600
C5A—H5A3	0.9600	C5C—H5C2	0.9600
C6A—H6A1	0.9600	C5C—H5C3	0.9600
C6A—H6A2	0.9600	C6C—H6C1	0.9600
C6A—H6A3	0.9600	C6C—H6C2	0.9600
O1B—C1B	1.249 (5)	C6C—H6C3	0.9600
			
O2C^i^—Mn1—O2C	180.0	C1B—O1B—Mn1	135.7 (3)
O2C^i^—Mn1—O1B	86.19 (12)	C1B—O2B—Mn2	124.0 (3)
O2C—Mn1—O1B	93.81 (12)	O1B—C1B—O2B	127.1 (5)
O2C^i^—Mn1—O1B^i^	93.81 (12)	O1B—C1B—C2B	119.7 (4)
O2C—Mn1—O1B^i^	86.19 (12)	O2B—C1B—C2B	113.1 (4)
O1B—Mn1—O1B^i^	180.0	N3B—C2B—C1B	117.9 (4)
O2C^i^—Mn1—O1A^ii^	88.25 (12)	N3B—C2B—H2B1	104 (3)
O2C—Mn1—O1A^ii^	91.75 (12)	C1B—C2B—H2B1	107 (3)
O1B—Mn1—O1A^ii^	93.30 (13)	N3B—C2B—H2B2	105 (5)
O1B^i^—Mn1—O1A^ii^	86.70 (13)	C1B—C2B—H2B2	113 (5)
O2C^i^—Mn1—O1A^iii^	91.75 (12)	H2B1—C2B—H2B2	109 (5)
O2C—Mn1—O1A^iii^	88.25 (12)	C4B—N3B—C6B	109.3 (5)
O1B—Mn1—O1A^iii^	86.70 (13)	C4B—N3B—C5B	107.8 (5)
O1B^i^—Mn1—O1A^iii^	93.30 (13)	C6B—N3B—C5B	108.5 (5)
O1A^ii^—Mn1—O1A^iii^	180.0	C4B—N3B—C2B	113.8 (4)
O1C^ii^—Mn2—O1C	180.0	C6B—N3B—C2B	109.1 (4)
O1C^ii^—Mn2—O2B	90.79 (12)	C5B—N3B—C2B	108.2 (4)
O1C—Mn2—O2B	89.21 (12)	N3B—C4B—H4B1	109.5
O1C^ii^—Mn2—O2B^ii^	89.21 (12)	N3B—C4B—H4B2	109.5
O1C—Mn2—O2B^ii^	90.79 (12)	H4B1—C4B—H4B2	109.5
O2B—Mn2—O2B^ii^	180.0	N3B—C4B—H4B3	109.5
O1C^ii^—Mn2—O2A^ii^	91.45 (12)	H4B1—C4B—H4B3	109.5
O1C—Mn2—O2A^ii^	88.55 (12)	H4B2—C4B—H4B3	109.5
O2B—Mn2—O2A^ii^	86.67 (12)	N3B—C5B—H5B1	109.5
O2B^ii^—Mn2—O2A^ii^	93.33 (12)	N3B—C5B—H5B2	109.5
O1C^ii^—Mn2—O2A	88.55 (12)	H5B1—C5B—H5B2	109.5
O1C—Mn2—O2A	91.45 (12)	N3B—C5B—H5B3	109.5
O2B—Mn2—O2A	93.33 (12)	H5B1—C5B—H5B3	109.5
O2B^ii^—Mn2—O2A	86.67 (12)	H5B2—C5B—H5B3	109.5
O2A^ii^—Mn2—O2A	180.0	N3B—C6B—H6B1	109.5
Br2—Mn3—Br4	110.42 (4)	N3B—C6B—H6B2	109.5
Br2—Mn3—Br1	113.26 (4)	H6B1—C6B—H6B2	109.5
Br4—Mn3—Br1	108.64 (4)	N3B—C6B—H6B3	109.5
Br2—Mn3—Br3	108.51 (4)	H6B1—C6B—H6B3	109.5
Br4—Mn3—Br3	108.99 (5)	H6B2—C6B—H6B3	109.5
Br1—Mn3—Br3	106.89 (4)	C1C—O1C—Mn2	124.6 (3)
C1A—O1A—Mn1^iv^	138.8 (3)	C1C—O2C—Mn1	136.7 (3)
C1A—O2A—Mn2	123.0 (3)	O1C—C1C—O2C	126.7 (4)
O1A—C1A—O2A	127.0 (4)	O1C—C1C—C2C	113.7 (4)
O1A—C1A—C2A	120.5 (4)	O2C—C1C—C2C	119.5 (4)
O2A—C1A—C2A	112.5 (4)	N3C—C2C—C1C	117.5 (4)
N3A—C2A—C1A	119.0 (4)	N3C—C2C—H2C1	106 (3)
N3A—C2A—H2A1	104 (3)	C1C—C2C—H2C1	109 (3)
C1A—C2A—H2A1	110 (3)	N3C—C2C—H2C2	108 (3)
N3A—C2A—H2A2	110 (4)	C1C—C2C—H2C2	108 (3)
C1A—C2A—H2A2	106 (4)	H2C1—C2C—H2C2	108 (4)
H2A1—C2A—H2A2	108 (5)	C5C—N3C—C4C	108.2 (5)
C4A—N3A—C6A	110.1 (6)	C5C—N3C—C6C	109.6 (4)
C4A—N3A—C2A	110.4 (5)	C4C—N3C—C6C	108.0 (4)
C6A—N3A—C2A	111.7 (5)	C5C—N3C—C2C	110.5 (4)
C4A—N3A—C5A	110.2 (6)	C4C—N3C—C2C	108.4 (4)
C6A—N3A—C5A	106.9 (5)	C6C—N3C—C2C	112.0 (4)
C2A—N3A—C5A	107.4 (4)	N3C—C4C—H4C1	109.5
N3A—C4A—H4A1	109.5	N3C—C4C—H4C2	109.5
N3A—C4A—H4A2	109.5	H4C1—C4C—H4C2	109.5
H4A1—C4A—H4A2	109.5	N3C—C4C—H4C3	109.5
N3A—C4A—H4A3	109.5	H4C1—C4C—H4C3	109.5
H4A1—C4A—H4A3	109.5	H4C2—C4C—H4C3	109.5
H4A2—C4A—H4A3	109.5	N3C—C5C—H5C1	109.5
N3A—C5A—H5A1	109.5	N3C—C5C—H5C2	109.5
N3A—C5A—H5A2	109.5	H5C1—C5C—H5C2	109.5
H5A1—C5A—H5A2	109.5	N3C—C5C—H5C3	109.5
N3A—C5A—H5A3	109.5	H5C1—C5C—H5C3	109.5
H5A1—C5A—H5A3	109.5	H5C2—C5C—H5C3	109.5
H5A2—C5A—H5A3	109.5	N3C—C6C—H6C1	109.5
N3A—C6A—H6A1	109.5	N3C—C6C—H6C2	109.5
N3A—C6A—H6A2	109.5	H6C1—C6C—H6C2	109.5
H6A1—C6A—H6A2	109.5	N3C—C6C—H6C3	109.5
N3A—C6A—H6A3	109.5	H6C1—C6C—H6C3	109.5
H6A1—C6A—H6A3	109.5	H6C2—C6C—H6C3	109.5
H6A2—C6A—H6A3	109.5		

Symmetry codes: (i) −*x*+2, −*y*, −*z*+2; (ii) −*x*+1, −*y*, −*z*+2; (iii) *x*+1, *y*, *z*; (iv) *x*−1, *y*, *z*.

## References

[bb1] Bergerhoff, G., Berndt, M. & Brandenburg, K. (1996). *J. Res. Natl Inst. Stand. Technol.* **101**, 221–225. 10.6028/jres.101.023 PMC4963142 27805159

[bb2] Bruker (2004). *SMART* and *SAINT-Plus* Bruker AXS Inc., Madison, Wisconsin, USA.

[bb3] Chen, X.-M. & Mak, T. C. W. (1991). *Inorg. Chim. Acta*, **189**, 3–5.

[bb4] Chen, X.-M. & Mak, T. C. W. (1994). *Inorg. Chem.* **33**, 2444–2447.

[bb5] Haussühl, S. (1988). *Solid State Commun.* **68**, 963–966.

[bb6] Haussühl, S. (1989). *Z. Kristallogr.* **188**, 311–320.

[bb7] Haussühl, E. & Schreuer, J. (2001). *Z. Kristallogr.* **216**, 616–620.

[bb8] Haussühl, S. & Wang, J. (1989). *Z. Kristallogr.* **187**, 249–251.

[bb9] Mak, T. C. W. (1990). *J. Mol. Struct.* **220**, 13–18.

[bb10] Otwinowski, Z. & Minor, W. (1997). *Methods in Enzymology*, Vol. 276, *Macromolecular Crystallography*, Part A, edited by C. W. Carter Jr & R. M. Sweet, pp. 307–326. New York: Academic Press.

[bb11] Schreuer, J. & Haussühl, S. (1993). *Z. Kristallogr.* **205**, 309–310.

[bb12] Sheldrick, G. M. (2008). *Acta Cryst.* A**64**, 112–122. 10.1107/S0108767307043930 18156677

[bb13] Viertorinne, M., Valkonen, J., Pitkänen, I., Mathlouthi, M. & Nurmi, J. (1999). *J. Mol. Struct.* **477**, 23–29.

[bb14] Wang, J., Gnanam, F. D. & Haussühl, S. (1986). *Z. Kristallogr.* **175**, 155–158.

[bb15] Wiehl, L., Schreuer, J. & Haussühl, E. (2006*a*). *Z. Kristallogr. New Cryst. Struct.* **221**, 77–79.

[bb16] Wiehl, L., Schreuer, J., Haussühl, E., Winkler, B., Removic-Langer, K., Wolf, B., Lang, M. & Milman, V. (2006*b*). *J. Phys. Condens. Matter*, **18**, 11067–11079.

